# Three-step management of a newborn with a giant, highly vascularized, cervical teratoma: a case report

**DOI:** 10.1186/s13256-019-1976-0

**Published:** 2019-03-10

**Authors:** Ori Hochwald, Ziv Gil, Arie Gordin, Zeev Winer, Ron Avrahami, Eitan Abargel, Asaad Khoury, Amit Lehavi, Philippe Abecassis, Liron Eldor, Ofer Ben-Izhak, Liron Borenstein-Levin, Ran Stienberg, Amir Kugelman

**Affiliations:** 1 Neonatal Intensive Care Unit, Ruth Rappaport Children’s Hospital, Rambam Health Campus, Haifa, Israel; 2 Department of Otolaryngology Head and Neck Surgery, Rambam Health Campus, Haifa, Israel; 3 The Pediatric ENT service, Rambam Health Campus, Haifa, Israel; 4 The Obstetrics & Gynecology Division, Rambam Health Campus, Haifa, Israel; 5 Invasive Neuroradiology Unit, Rambam Health Campus, Haifa, Israel; 6 Department of Pediatric Cardiology & Congenital Heart Disease in Adults, Ruth Rappaport Children’s Hospital, Rambam Health Campus, Haifa, Israel; 7 The Department of Anesthesiology, Rambam Health Campus, Haifa, Israel; 8 The Department of Plastic Surgery, Rambam Health Campus, Haifa, Israel; 9 The Department of Pathology, Rambam Health Campus, Haifa, Israel; 10 The Department of Pediatric Surgery, Rambam Health Campus, Haifa, Israel

**Keywords:** Newborn, Congenital, Cervical, Teratoma, Endovascular embolization, *Ex utero* intrapartum treatment

## Abstract

**Background:**

A giant congenital cervical teratoma is often highly vascularized; thus, in addition to a life-threatening airway occlusion at birth it comprises a high risk for significant and lethal blood loss during resection. In the case presented, an endovascular embolization of the carotid artery that supplied a giant congenital cervical teratoma was done as part of a three-stage treatment soon after birth and contributed to an overall good outcome. Embolization in cases of cervical teratomas was not described previously.

**Case presentation:**

We present a case of a preterm newborn from a Sephardic jewish origin with a giant, highly vascularized, congenital cervical teratoma that was managed successfully in three stages: (1) delivery by an *ex utero* intrapartum treatment procedure after extensive preoperative planning and followed by tracheostomy, (2) endovascular embolization of the carotid artery that supplied the tumor in order to decrease blood loss during resection, and (3) complete surgical resection. The parents were involved in all the ethical and medical decisions, starting just after the cervical mass was diagnosed prenatally.

**Conclusion:**

The management of giant congenital cervical teratoma is often challenging from both a medical and ethical prospective. Meticulous perinatal planning and parents’ involvement is crucial. Endovascular embolization of the tumor feeding vessels can significantly improve the resection outcome and overall prognosis.

## Introduction

Congenital teratomas (from the Greek “*téras oma*” meaning “monstrous tumor”) are rare tumors (1 per 40,000 births [1]) containing tissues derived from all three primordial embryonic layers [2]. The most common site is the sacrococcygeal region. Less than 10% of all congenital teratomas are located in the neck [2, 3]. Although these lesions are usually histologically benign, they are usually large, and when they are located in the neck, perinatal mortality can be high as a result of upper airway obstruction [3]. Once the airway is secured, the resection of a highly vascularized, congenital giant teratoma can still be a fatal procedure due to massive bleeding of the tumor [4].

The pathophysiology of teratoma remains unknown. Neither maternal age nor specific ethnicities were found to be associated with this tumor. In some cases, genetic changes in the tumor cells were demonstrated and include 1p21.1 amplification, 9p22 deletion, and 17q21.33 1-copy gain [5]. Associated malformations are rare and include gastrointestinal (for example, imperforate anus), cardiac (for example, hypoplastic left heart syndrome), and neurological (for example, absence of corpus callosum and arachnoid cysts) defects [1].

Prenatal diagnosis, anticipation of neonatal upper airway obstruction, delivery by *ex utero* intrapartum treatment (EXIT) procedure, and multidisciplinary team care can result in improved perinatal outcome [6]. We present a case of a highly vascularized, giant congenital cervical teratoma that was managed successfully in three stages: (1) delivery by an EXIT procedure after extensive preoperative planning and followed by tracheostomy, (2) endovascular embolization of the carotid artery that supplied the tumor in order to decrease blood loss during resection, and (3) complete surgical resection. The parents were involved in all the ethical and medical decisions, starting before the delivery, just after the diagnosis of the cervical mass.

## Case presentation

An otherwise healthy 33-year-old woman, gravida 3, para 2, from a Sephardic Jewish origin, was initially referred to our institution at 30.6 weeks of gestation due to a large neck mass found on prenatal ultrasonography (US). Her previous two pregnancies were uncomplicated. The fetal sonogram showed a 10 by 8 cm mass on the right side of the neck, which was not present in detailed scans taken at 14 and 22 weeks. The mass was composed of a cystic portion and a solid portion containing blood vessels and was growing rapidly in subsequent ultrasound studies. A significant polyhydramnios with amniotic fluid index (AFI) of 50 suggested an upper gastrointestinal obstruction and a highly possible airway obstruction as well. Findings were confirmed by fetal magnetic resonance imaging (MRI). In anticipation of the difficulty in establishing a secured airway at birth and the potential complicated resection of the giant tumor after birth, the mother was referred to our hospital for consultation.

The parents were in consultation with the maternal fetal team, neonatologist, anesthesiologist, pediatric surgeon, and otolaryngologist. The parents were presented with a guarded prognosis but insisted that the pregnancy continue with maximal efforts during delivery and during the neonatal period.

Therefore, a planned EXIT procedure, which provides the best chance to establish a patent airway, was offered to our patient, presenting the risks [7]. Specifically, we informed the parents about the risks for the mother, including significant hemorrhage from the uterus due to the uterine relaxation necessary to avoid placental separation, with a possible uterine resection in the case of a life-threatening hemorrhage.

Knowing the risk of an unplanned preterm delivery due to polyhydramnios and uterine contractions, we scheduled our patient for a planned cesarean delivery at 34 weeks organizing and preparing a multidisciplinary team ready to perform the EXIT procedure.

### The EXIT procedure

A multidisciplinary team including obstetricians, anesthesiologists, neonatologists, otolaryngologists, pediatric surgeons, pulmonologists, cardiologists, and nursing staff participated. A combined epidural and general anesthesia was planned. Our patient’s blood pressure was monitored continuously to detect and treat a possible event of maternal hypotension in order to maintain good fetal perfusion. After epidural catheter placement while lying on her left side, the parturient returned to lie on her back. Immediately, a severe hypotension (65/30 mmHg) with tachycardia (150 beats/minute) appeared. We related this complication to the polyhydramnios causing a significant decrease in the vena cava flow. After left uterine displacement and bolus of phenylephrine, her blood pressure and heart rate returned to normal. General anesthesia with rapid sequence induction was induced with succinylcholine (100 mg) and propofol (150 mg). During the EXIT procedure an appropriate uterine relaxation was maintained to prevent placenta expulsion. We used a high dose of inhaled anesthetics and minimal nitroglycerine intravenous drip. No bleeding occurred during the procedure.

A classical uterine incision was made and only the fetal head and upper chest with the cervical mass were delivered through the uterine incision. The rest of the body and the cord were left *in situ* to avoid placental separation. The amniotic fluid was slowly drained to avoid an abrupt drainage of the fluid and an early separation of the placenta.

The multidisciplinary team had planned and rehearsed the following escalating step-by-step scenarios for establishing a secured airway: (1) direct laryngoscopy and intubation attempt by a pediatric anesthesiologist with the aid of a neonatologist, (2) rigid bronchoscopy by an otolaryngologist and possible aid of flexible bronchoscopy by a pediatric pulmonologist, (3) if laryngoscopy and bronchoscopy failed, a tracheostomy was planned by an otolaryngology team. As the tumor was highly vascularized, any debulking procedure was impractical and would have imminently put both the mother and newborn at the risk of death. Analgesia for the newborn using intramuscular fentanyl was prepared in advance.

**Fig. 1:**
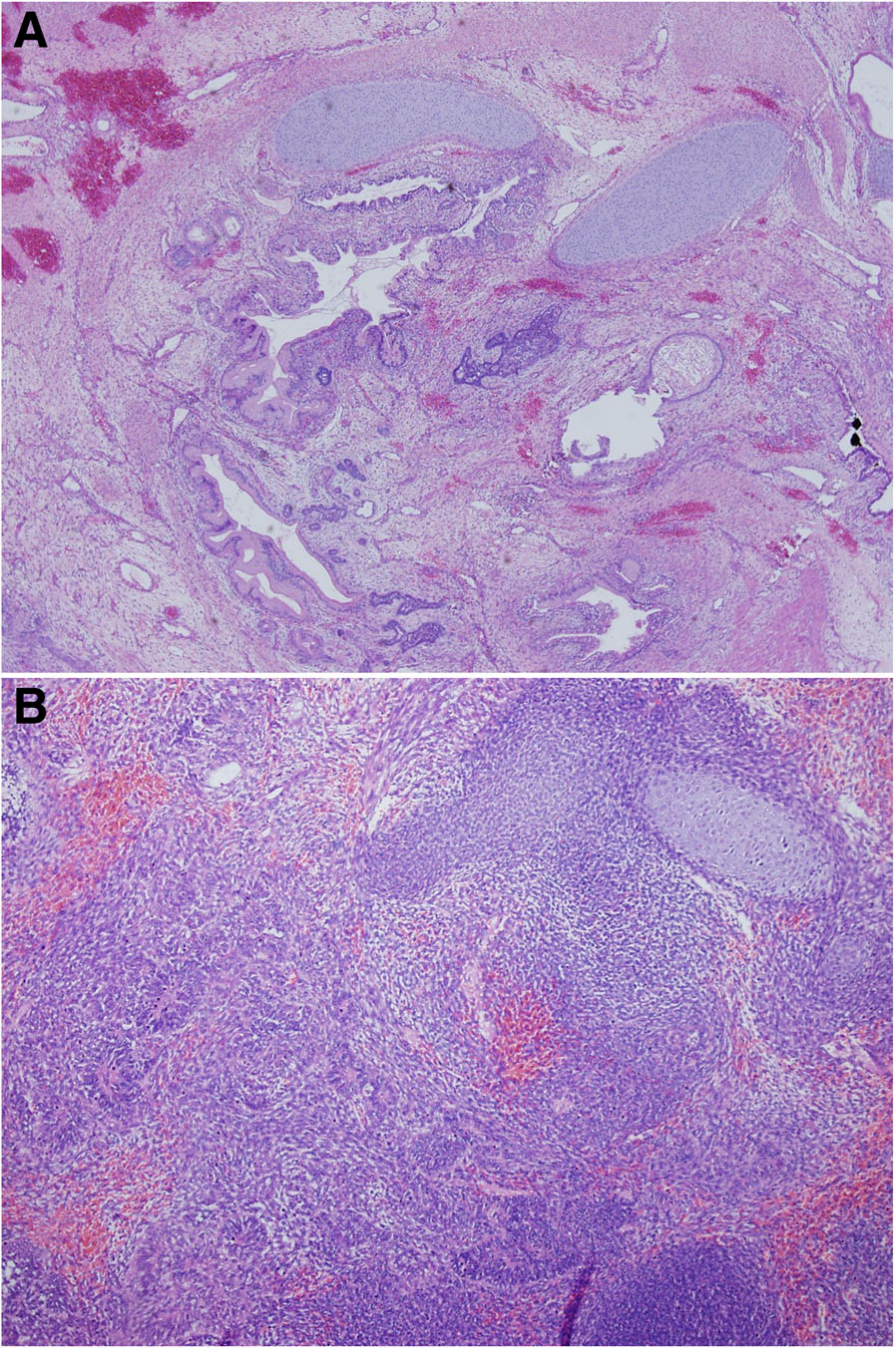
The tumor biopsy histology. **a** Teratoma containing mature epithelial elements, cartilage, and mesenchyme. **b** Immature teratoma containing neuroepithelium and primitive small round blue cells

After the head of the newborn emerged, a direct laryngoscopy was attempted; however, the larynx was not visible as the tumor obstructed the pharynx. Attempts at direct intubation were abolished after 1 minute. Next, a rigid bronchoscopy was performed and only the tip of the epiglottis was visualized. At 11 minutes from delivery, endotracheal intubation was successfully performed. From the time of delivery to the time of intubation, the presence of a good heart rate of the fetus was monitored by echocardiography. After the airway was secured, the female newborn was delivered, the cord was clamped, and the placenta extracted. The arterial cord pH was 7.01 with CO_2_ of 71 mmHg and lactate of 9.5 mmol/L. Immediately after delivery, a computed tomography (CT) scan was performed under general anesthesia that demonstrated that a tracheostomy could be performed without interfering with the tumor. During the tracheostomy, a biopsy was taken from the tumor that demonstrated both mature and immature teratoma (Fig. 1).

**Fig. 2:**
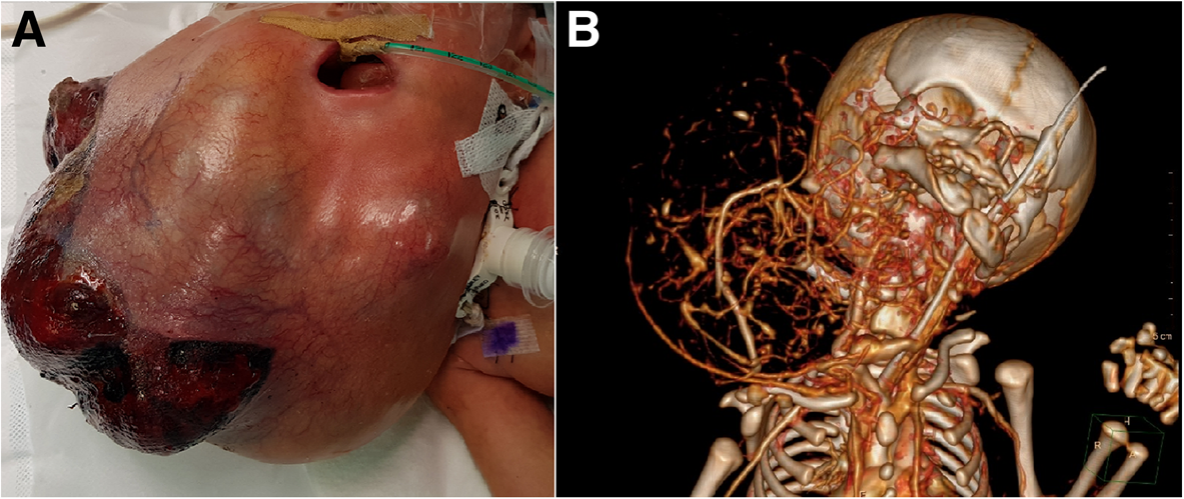
**a** The giant congenital teratoma on the third day of life. The tumor was covered with a thin skin layer with a large cystic and solid appearance. There were occasional bleedings from several lacerated areas on the tumor surface. **b** A computed tomography study demonstrated the abundant blood supply of the tumor including the fact that the right carotid artery was supplying this tumor

The birth weight of the newborn girl was 3 kg including the tumor. A giant submental mass that protruded from the right side of her neck was noted. The tumor was covered with a thin skin layer with a large cystic and solid appearance. There were occasional bleedings from several lacerated areas on the tumor surface (Fig. 2a). A CT study demonstrated the abundant blood supply of the tumor including the fact that the right carotid artery was supplying this tumor (Fig. 2b). During the first 2 days of life, before the next procedure, the tumor continued to grow significantly, probably partially due to internal bleeding. Her heart function was normal but prerenal azotemia evolved due to loss of large amounts of serotic fluid as well as blood from the lacerated mass.

### Endovascular embolization

**Fig. 3:**
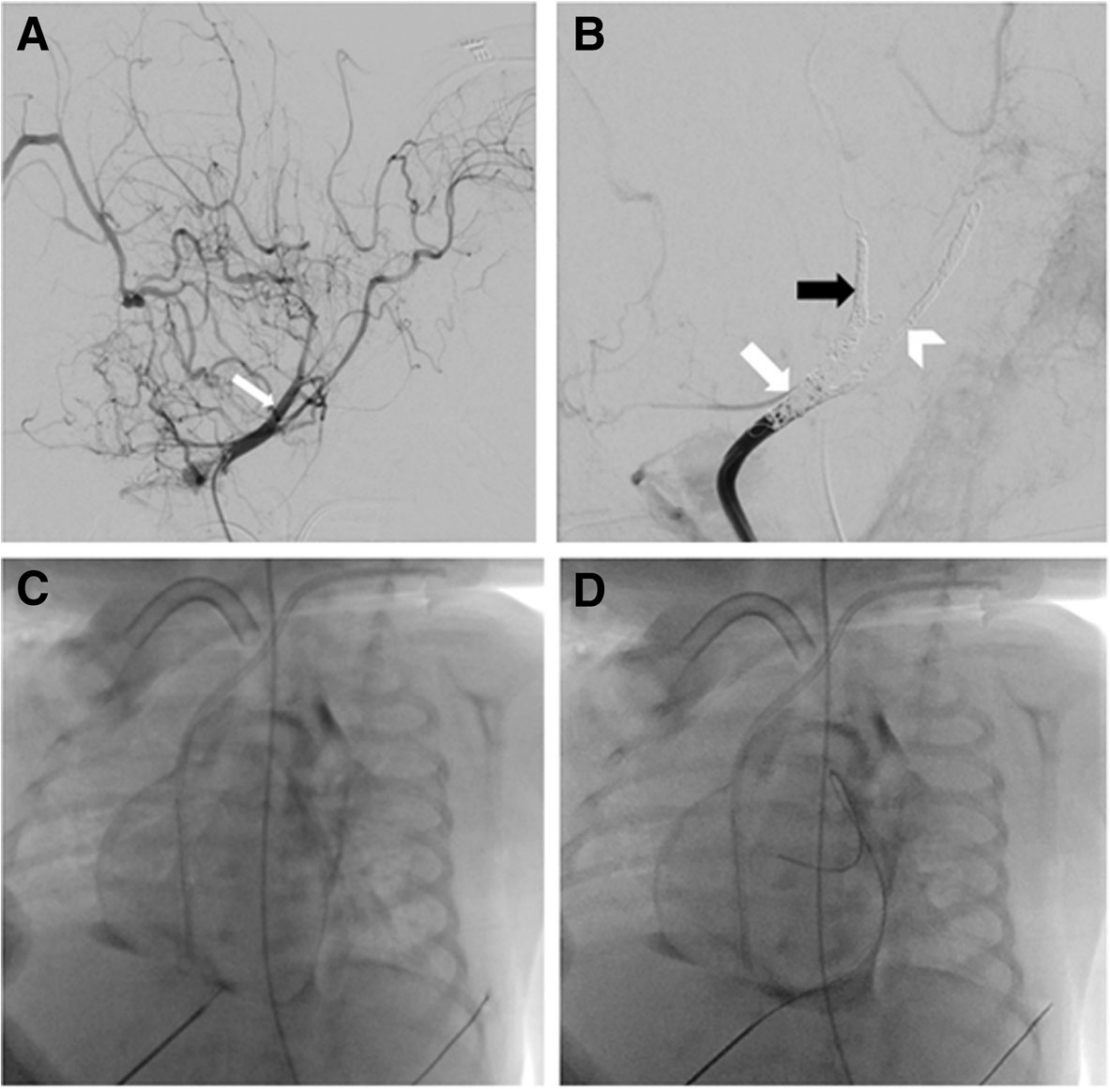
**a** Pretreatment lateral view of the right common carotid, notice the large and rich vascularization of the tumor from the external carotid branches (*white arrow*). **b** Post embolization lateral view, platinum coils in common carotid (*white arrow*), internal carotid (*arrow head*), and external carotid (*black arrow*); notice massive reduction in tumor vascularization. **c** Contrast medium in the pericardial space. **d** Pericardiocentesis wire in the pericardial space

Because resection of the giant, highly vascularized, cervical teratoma could be a fatal procedure due to massive bleeding, we used endovascular embolization a day prior to the tumor resection. The embolization was done by a pediatric cardiologist and an interventional neuroradiologist. Cervical angiography via a femoral line catheter demonstrated the highly vascularized tumor supplied by the external right carotid artery (Fig. 3a). A carotid occlusion test was performed and showed good blood supply from the contralateral internal carotid artery (ICA). Next, the origin of the external carotid was occluded using detachable platinum coils. Since resection of the tumor implied the sacrifice of all cervical carotid branches it was mandatory to also occlude the cervical ICA and common carotid artery to achieve minimal blood loss during surgery (Fig. 3b). The procedure was done under general anesthesia.

Upon withdrawal of the angiography catheter a sudden deterioration of our patient was noted. Fluoroscopy and echocardiography demonstrated cardiac tamponade, probably from a small laceration in the aorta. An urgent pericardiocentesis retrieving 3 ml of blood from the pericardial space allowed fast and full hemodynamic recovery (Fig. 3c, d). There was no re-accumulation of pericardial blood.

### The tumor resection

**Fig. 4:**
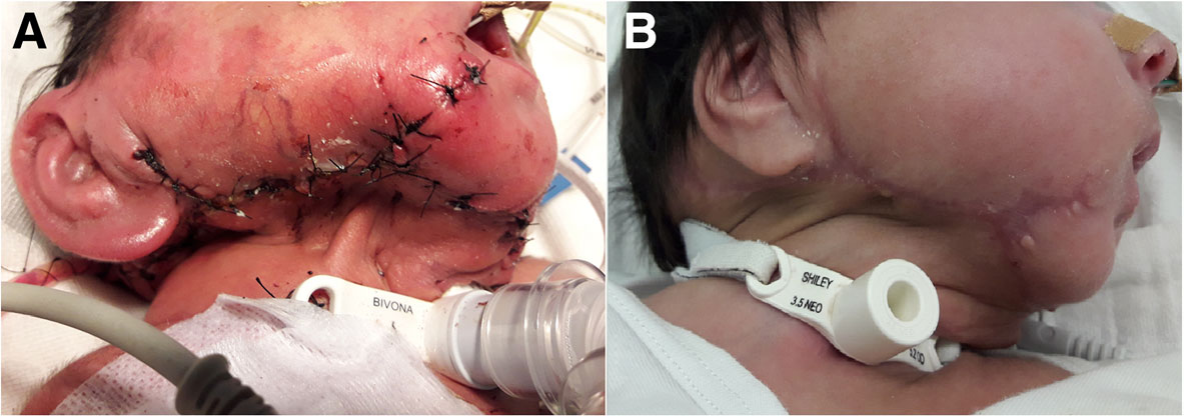
**a** One day after the resection. **b** Before discharge, at 3 months of age

Less than 24 hours after the embolization**,** the surgical team, which included a head and neck surgeon, pediatric otolaryngologist, and a plastic surgeon, performed the surgery with a pediatric anesthesiologist. Using LigaSure™ Sealer/Divider (Medtronic), the tumor was dissected including a section of the lacerated skin. Because the main blood supply of the tumor was embolized, the surgery was performed from its distal part along our patient’s mandible towards the proximal part at the junction of her neck and thorax. The tumor impinged into her pharynx, and her larynx and epiglottis were identified and preserved. Her mandible and neck muscles were preserved; however, her vagal nerve was surrounded by the tumor and was sacrificed with the carotid artery. The tumor weighted 800 grams and the overall blood loss was 300 ml. After tumor resection and closure of the skin (Fig. 4), the tracheostomy tube was changed. This was followed by a pneumothorax that required a chest tube insertion.

### After the procedure

Gradually, after the tumor resection, our patient was weaned from mechanical ventilation. She was fed initially using an orogastric tube and gradually learned to feed orally. After the resection, a right vocal cord paralysis was observed using a flexible fiberoptic laryngoscopy; therefore, we decided to leave the tracheal tube in place. We attributed this finding to an injury of the recurrent laryngeal nerve during the resection. Another neurological sequela was an abduction weakness of her right shoulder which slowly recovered. Neck teratomas can arise from and completely replace the thyroid tissue [1]. Thyroid function tests demonstrated hypothyroidism and she started receiving thyroid replacement therapy. A follow-up ultrasound of her head was normal. A brain MRI done a month after the final surgery demonstrated normal brain appearance. She was discharged to her home at 3 months of age (Fig. 4).

## Discussion

Cervical teratomas can acquire gigantic dimensions in late gestation. The compression over the esophagus causes polyhydramnios and the associated compression over the trachea can cause airway obstruction at birth.

Severe polyhydramnion may lead to preterm delivery, thus complicating the upper airway obstruction with immature lungs and respiratory drive. Planning secure resuscitation at birth needs to take into account the availability of multidisciplinary teams and the issue of prematurity; thus finding the best timing for intervention.

EXIT is a surgical delivery procedure used to deliver fetuses who have airway compression. Failure to achieve airway under controlled and safe conditions and in a reasonable time might result in asphyxia.

Giant congenital cervical teratomas are challenging lesions. After making the *in utero* diagnosis, and following thorough assessment and consultations, our team felt that the risk for the mother during the EXIT procedure and the risks and possible sequelae for the infant during and after the different procedures after birth were significant enough that offering no treatment was ethically reasonable. The parents, fully aware of all options and risks, decided on maximal care to their daughter.

## Conclusions

In the case of our patient, once stabilized with tracheostomy after EXIT, we found the tumor to have a highly abundant blood supply. Although pre-excision embolization was described sporadically in cases of sacral teratoma [4, 8], we found no literature on embolization in cases of cervical teratomas. We assumed that embolization prior to definite surgical resection of the tumor could decrease significantly the blood loss and risk for complications or death during the procedure.

The clinical challenges reported in this case highlight the importance of multidisciplinary teams in the management of congenital cervical tumors. This case also emphasizes the ethical issues and the need for an open discussion with the parents in a similar life-threatening prenatal diagnosis.

## Abbreviations

CT: Computed tomography
EXIT: *Ex utero* intrapartum treatment
ICA: Internal carotid artery
MRI: Magnetic resonance imaging
US: Ultrasonography
